# Post-Operative Assessment of Ulnar Nerve Tension Using Shear-Wave Elastography

**DOI:** 10.3390/neurolint13030046

**Published:** 2021-09-14

**Authors:** Sebastien Durand, Wassim Raffoul, Thierry Christen, Nadine Pedrazzi

**Affiliations:** Department of Hand Surgery, Lausanne University Hospital, Rue du Bugnon 46, CH-1011 Lausanne, Switzerland; Wassim.Raffoul@chuv.ch (W.R.); thierry.christen@chuv.ch (T.C.); nadine.pedrazzi@chuv.ch (N.P.)

**Keywords:** elastography, ulnar nerve, nerve transposition, nerve compression

## Abstract

Background: Ulnar nerve compression at the elbow level is the second-most common entrapment neuropathy. The aim of this study was to use shear-wave elastography for the quantification of ulnar nerve elasticity in patients after ulnar nerve decompression with anterior transposition and in the contralateral non-operative side. Method: Eleven patients with confirmed diagnosis and ulnar nerve decompression with anterior transposition were included and examinations were performed on an Aixplorer^TM^ ultrasound system (Supersonic Imagine, Aix-en-Provence, France). Results: We observed significant differences at 0-degree (*p* < 0.001), 45-degree (*p* < 0.05), 90-degree (*p* < 0.01) and 120-degree (*p* < 0.001) elbow flexion in the shear elastic modulus of the ulnar nerve in the operative and non-operative sides. There were no statistically significant differences between the elasticity values of the ulnar nerve after transposition at 0-degree elbow flexion and in the non-operative side at 120-degree elbow flexion (*p* = 0.39), or in the ulnar nerve after transposition at 120-degree elbow flexion and in the non-operative side at 0-degree elbow flexion (*p* = 0.09). Conclusion: Shear-wave elastography has the potential to be used postoperatively as a method for assessing nerve tension noninvasively by the estimation of mechanical properties, such as the shear elastic modulus.

## 1. Introduction

With an annual incidence of 24.7 per 100,000 [1], cubital tunnel syndrome is the second-most common entrapment neuropathy after carpal tunnel syndrome [1,2,3,4,5,6,7]. Its etiology is mostly idiopathic, although it is favored by repetitive elbow flexion, repetitive trauma, disproportionate strain, snapping triceps syndrome, degenerative arthritis and space-occupying lesions [3]. Ulnar neuropathy is identified by clinical history, physical examination and electroneuromyography (ENMG), which represents a key component in this diagnosis [2,8]. For mild cases, non-surgical management is indicated [4,9].

Surgical decompression of the ulnar nerve is indicated in cases recalcitrant to conservative management and in patients who initially present a more severe neuropathy. One of two techniques is generally proposed: simple release of the ulnar nerve or neurolysis and anterior transposition [4]. Ulnar nerve release, with transposition from posterior to anterior to the axis of rotation of the elbow, reduces the tension on the nerve in elbow flexion. This technique has been considered in patients with ulnar nerve instability [4], severe osteoarthritis of the elbow, cubitus valgus and the presence of a mass within the cubital tunnel. Numerous controlled trials comparing in situ decompression and neurolysis with transposition did not find any differences in outcome, with equivalent neurophysiological and clinical improvement [7,9].

Shear-wave elastography (SWE) is a recently developed ultrasound imaging technology that provides quantitative values of soft tissue stiffness, including in the muscle, tendon and nerve. It improves the diagnostic performance of ultrasound imaging for various neuropathies involving the median, sciatic, tibial and ulnar nerves [10]. This technique may find application for surgeons as an instrument to detect when peripheral nerves are exposed to mechanical overload after nerve transposition or end-to-end neurorrhaphy. It may also allow the comparison of patient outcomes after surgery and improve surgical procedures. However, to the best of our knowledge, the in vivo mechanical outcome of ulnar nerve transposition has not been studied thus far. The aim of this study was to assess the kinematic profile of nerves after ulnar nerve transposition, and to use SWE for the quantification of ulnar nerve elasticity related to nerve tension in patients after ulnar nerve decompression with anterior transposition and in the healthy contralateral side.

## 2. Material and Methods

### 2.1. Subjects

Written consent was obtained from all patients. This retrospective chart review study involving human participants was conducted in accordance with the ethical standards of the institutional and national research committee and with the 1964 Helsinki Declaration and its later amendments or comparable ethical standards. The study included patients who suffered from ulnar nerve compression on the cubital tunnel diagnosed by electromyography or echography. They underwent ulnar nerve decompression with anterior transposition at our institution between 2018 and 2020. Patients with symptoms of the contralateral elbow, or without ultrasonographic imaging, were excluded.

### 2.2. Operative Procedure

**Figure 1:**
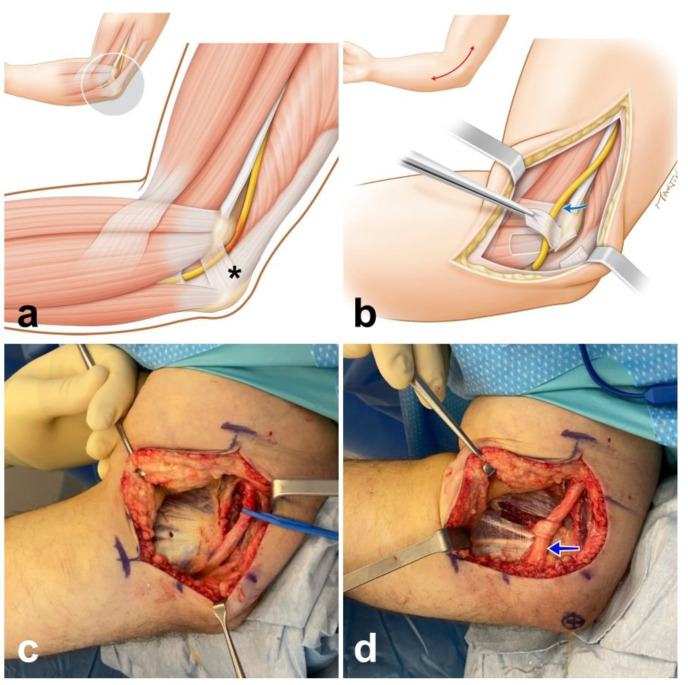
Sketches detailing ulnar nerve (yellow) entrapment in the cubital tunnel (**a**), which is where Obsorne’s ligament is located (asterix). The ulnar nerve is transposed anteriorly (blue arrow) and stabilized with a fascial sling (**b**). Intraoperative photograph after complete release of all points of ulnar nerve with elevation of the soft tissue above the flexor-pronator muscle mass (**c**); the ulnar nerve is transposed subcutaneously and stabilized anteriorly with a fascial sling (**d**).

The procedure was performed under axillary nerve block and tourniquet. A 12–15 cm longitudinal incision was made posteriorly to the medial epicondyle. Complete release of all points of ulnar nerve compression was performed: the Osborne’s ligament, the aponeurosis of the flexor carpi ulnaris, the medial intermuscular septum and the tunnel outlet between the two muscle heads of flexor carpi ulnaris [3]. Care was taken to identify and protect branches of the medial antebrachial cutaneous nerve. The soft tissue above the flexor-pronator muscle mass was elevated, and the ulnar nerve was transposed subcutaneously and stabilized anteriorly using a fascial sling [4,9] (Figure 1).

### 2.3. Ultrasound Protocol

Ulnar nerve ultrasonographic examination was performed postoperatively in all included patients in the operated elbow and the contralateral non-operated healthy side. Given the retrospective design of the study, preoperative nerve examinations were not possible.

Examinations were performed on an Aixplorer™ ultrasound system (Supersonic Imagine, Aix-en-Provence, France). A high-resolution 5–18 MHz linear array transducer (SuperLinear™ SL18-5; Supersonic Imagine, Aix-en-Provence, France) with 256 elements and a bandwidth of 5 to 18 Mhz was used with contact gel (Ecoultragel Pirrone & Co., Milan, Italy). Care was taken to avoid unnecessary pressure of the transducer on the skin.

**Figure 2:**
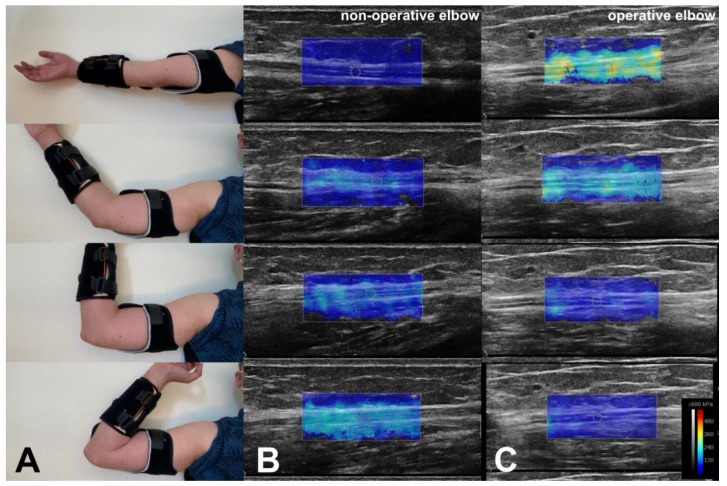
Examples of shear elastic modulus measurement of the ulnar nerve at 0- (image at top), 45-, 90- and 120-degree flexion of the elbow in an Elbo™ brace (**A**). The colored region represents the shear elasticity map in a healthy ulnar nerve (**B**) and an ulnar nerve after anterior transposition (**C**) with a color scale (see bottom right), with blue representing lesser stiffness and red greater stiffness. The software allowed us to measure the mean shear elasticity value over a 2 mm^2^ Q-Box focus area.

In B-mode, the ulnar nerve’s morphology was evaluated in the transverse plane via the honeycomb-like structure. The transducer was then rotated 90 degrees onto the longitudinal plane, and nerves were assessed by their straight hyperechoic boundaries and dissociated from vasculature using Doppler ultrasound [11]. Quantitative assessment of the nerve location according to the medial epicondyle was performed on the transverse plane. SWE with a 2 mm^2^ Q-Box (quantitative box) focus area was performed on the ulnar nerve in the longitudinal plane just proximal to the medial epicondyle in the operated and non-operated elbow. Nerve stiffness was recorded in patients after ulnar nerve decompression with anterior transposition, and on the contralateral non-operative healthy side (Figure 2). The quantitative values of elasticity of the ulnar nerve were obtained in m.s^−1^ (shear-wave velocity) and in kPa (shear elastic modulus) at 0, 45, 90 and 120 degrees of elbow flexion in an Elbo™ brace (Orthoservice AG, Chiasso, Switzerland).

### 2.4. Statistical Analysis

The normal distribution of the study variables was tested with the Shapiro–Wilk test. Student’s *t*-test was used to compare the elasticity of the ulnar nerve for different related conditions: 0-, 45-, 90- and 120-degree flexion of the elbow, and between the operative and non-operative side. Statistical significance was set at *p* < 0.05.

## 3. Results

We reviewed the charts of 18 consecutive patients who underwent ulnar nerve decompression with anterior transposition in our institution between 2018 and 2020. Seven patients were excluded due to the lack of elastography imaging, the presence of symptoms of ulnar nerve compression on the contralateral side, or limited elbow range of motion. The study included 11 patients with a mean age of 53.2 years-old (SD 14.9 years-old)—6 males (54.5%) and 5 females (45.5%). According to McGowan’s staging system [12], six patients were classified as grade 2 (moderate) and two were grade 3 (severe). Preoperatively, clinical instability in the ulnar nerve was present in six cases (54.5%). Decompression and transposition of the ulnar nerve was performed on the left side in six cases and on the right side in five cases. Patient data are recorded in Table 1.

In B-mode, we observed the persistence of the anterior transposition of the ulnar nerve in all patients at a mean duration between surgery and SWE of 8 months (SD 5.8 months). On the operated side, the mean distance between the center of the ulnar nerve and the tip of the medial epicondyle was 1.22 cm (SD 0.5 cm). The ulnar nerve glided away from the elbow joint in all patients with flexion of the elbow from 0 to 120 degrees. On the healthy side, the ulnar nerve remained posterior to the medial epicondyle in all patients, and glided toward the joint when the elbow was flexed from 0 to 120 degrees.

**Figure 3:**
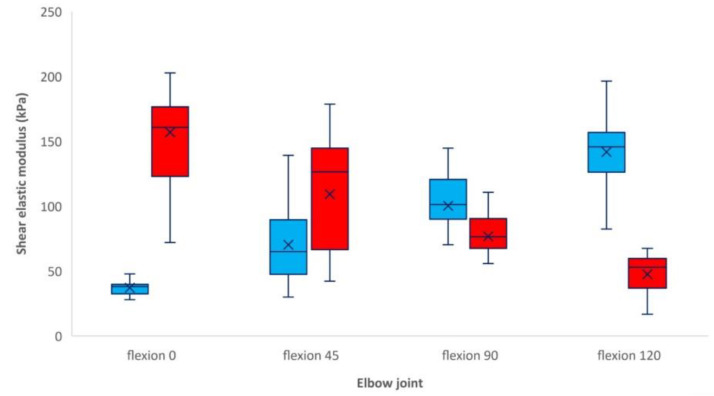
Box plot of the shear elastic modulus of the ulnar nerve (kPa) at 0, 45, 90 and 120 degrees of elbow flexion in operative side (red) and non-operative side (blue). Box (interquartile range (IQR); 75th–25th percentile), central line (median; 50th percentile), lower whisker (5th percentile), upper whisker (95th percentile).

We observed a significant difference in the shear elastic modulus of the ulnar nerve at 0 degrees (*p* < 0.001), 45 degrees (*p* < 0.05), 90 degrees (*p* < 0.01) and 120 degrees (*p* < 0.001) of elbow flexion between operative and non-operative sides. A comparable ulnar nerve elasticity value between both sides is expected between 45 and 90 degrees of elbow flexion (Figure 3).

In the non-operative side, we observed a significant differences in the shear elastic modulus between 0 degrees (mean 37.1 kPa, SDd 9.2 kPa) and 45 degrees of elbow flexion (mean 70.3 kPa, SD 32.1 kPa, *p* < 0.01), between 45 degrees and 90 degrees (mean 100.3 kPa, SD 29.4 kPa, *p* < 0.05), and between 90 degrees and 120 degrees (mean 141.7 kPa, SD 31.7 kPa, *p* < 0.005). On the operative side, we observed significant differences between 0 degrees (mean 157 kPa, SD 50.1 kPa) and 45 degrees (mean 109.1 kPa, SD 46.6 kPa, *p* < 0.01) and between 90 degrees (mean 76.7 kPa, SD 23.7 kPa) and 120 degrees (mean 47.4 kPa, SD 16.1 kPa, *p* < 0.05) of elbow flexion. No significant difference was observed between 45 degrees and 90 degrees. 

There were no statistically significant differences in shear elastic modulus between the ulnar nerve after transposition at 0 degrees of elbow flexion and the non-operative side at 120 degrees of elbow flexion (*p* = 0.39). There were no statistically significant differences in shear elastic modulus between the ulnar nerve after transposition at 120 degrees of elbow flexion and the non-operative side at 0 degrees of elbow flexion (*p* = 0.09).

## 4. Discussion

ENMG allows the functional analysis of peripheral nerve conduction, and currently represents the key diagnostic modality, providing details about the degree of myelin dysfunction and axonal loss [13]. However, this examination also presents some disadvantages, as it is time-consuming, can be painful, and carries a risk of false negative results [4,10]. Magnetic resonance imaging (MRI) is another helpful tool to evaluate soft tissues and determine the location of the injury, but it gives no indication about the severity of a neuropathy, and can only be used to confirm the diagnosis [4]. 

High-resolution ultrasound imaging has gained attention recently as a complementary investigatory tool of the peripheral nervous system, as many clinically important nerves are located superficially and are easily accessible for this type of examination [10,13,14]. Conventional B-mode ultrasound can provide information regarding the site of the lesion, the eventual loss of the normal fascicular architecture, abnormal nerve cross-sectional areas (CSAs) or echogenicity, increased vascularity, reduced or excessive nerve mobility, or anomalous anatomical structures causing nerve injury [8,9,10,11,12,13]. In several previous studies, patients with ulnar neuropathy at the elbow have been shown to have an increased CSA when compared with healthy control groups, but the cutoff above which CSA is considered pathological varies between authors [2,3,4,5,15,16]. 

It is now widely accepted that tissue stiffness is often correlated with pathological processes, such as the overload of connective tissue [17]. Neuropathies may cause a loss of myelinated axons and a subsequent increase in the intraneural connective tissue responsible for increasing nerve stiffness. Ulnar nerve compression results in a higher pressure in the cubital tunnel and causes ischemia, inflammation, edema and fibrosis in the intraneural space and synovium. This process results in increased nerve stiffness, which can be identified and monitored through SWE [11]. SWE can provide advanced quantitative data to assess soft tissue elasticity in ulnar entrapment neuropathy [2]. Therefore, patients with compressive neuropathy at the elbow have increased stiffness values in SWE, with the cutoffs varying from 31 kPa to 61 kPa [2,3,17]. Some authors believe that ultrasound, or more specifically SWE, will become part of the routine examination for the diagnosis of ulnar neuropathy at the elbow, in support of ENMG, or even alone [1,2,3,5,8,10,17,18]. 

The direction and magnitude of nerve excursion depend on the anatomical relationship between the nerve and the axis of rotation in the moving joint [11]. Our study demonstrates a statistically significant difference in the shear elastic modulus when considering the ulnar nerve’s location relative to the elbow’s axis of rotation. When the nerve bed is elongated, as in the case of 120° elbow flexion, the nerve is placed under increased tensile stress and glides toward the moving joint, a movement called convergence. If the nerve bed tension is relieved during elbow extension, the nerve glides away from the moving joint (divergence) [19]. This process seems to reverse following the anterior transposition of the ulnar nerve. Our study confirms the modification of the kinematic profile (convergence/divergence) of the ulnar nerve after anterior transposition, and demonstrates that there is a significant difference in the shear elastic modulus according to the degree of elbow flexion between the operative and non-operative sides. Analyses of the non-operated side showed a higher shear elastic modulus at the position of maximum elbow flexion (120° flexion) that decreased with elbow extension, with the lowest shear elastic modulus at 0° elbow flexion. Analyses of the operated side show a reversal of these data: the shear elastic modulus is highest with extension and decreases with elbow flexion. The shear elastic modulus of the transposed ulnar nerve at 0 degrees of elbow flexion was not statistically different from that of the nerve in the non-operative side at 120° elbow flexion (*p* = 0.39). Previous studies used cadaveric specimens and could only speculate on the mechanisms of elongation and excursion of the ulnar nerve after decompression and anterior transposition in the clinical setting [20]. After anterior transposition, the ulnar nerve section that was originally in the groove elongated (23%) with elbow extension to the same extent as the normal nerve during flexion [20].

It has been shown in vivo that shear-wave (SW) velocity is influenced by nerve tension, and the relationship between nerve SW velocity and stiffness cannot be isolated from the effects of forces acting upon the nerve [11]. SWE has the potential to be used as an additional noninvasive method for assessing nerve tension via the estimation of mechanical properties such as stiffness.

The ultrasonographic analysis and SWE monitoring of nerves might be of interest not only in the context of nerve transposition, but also after nerve sutures. While tensionless nerve suture has been considered standard practice for decades [21], some authors believe some amount of tension can sustain robust nerve regeneration [22]. This tension has been measured invasively in vivo and is between 0.39 and 0.56 N [23]. SWE may help to noninvasively determine a threshold of the shear elastic modulus at the nerve suture that is compatible with functional restoration. Further studies are needed to achieve this purpose.

Some limitations of this study must be mentioned. The number of patients is limited and the design of this analysis is retrospective. Elastography findings are highly dependent on the examiner, and mainly on the pressure exerted on the skin by the transducer. Every effort was made to apply the least pressure possible. Great variability in the measurement of the shear elastic modulus was observed. This can be explained by the variation in position between the transposed ulnar nerve and the medial epicondyle (Table 1), and by the variability in time after surgery (2–16 months). In addition, when a nerve is elongated, there is a rapid reduction in the stress within the nerve. If a measurement is not taken at exactly the same time for each patient, variability in the shear elastic modulus can be observed.

## 5. Conclusions

SWE is currently tested mostly as an additional tool in the evaluation of peripheral nerve disorders. It has shown utility compared to conventional ultrasound studies but requires further evaluation. Our results support previous findings that SWE has the potential to be used as an additional method for assessing nerve tension noninvasively by estimation of mechanical properties such as shear elastic modulus.

## Figures and Tables

**Table 1:** Patient demographics and clinical data, ENMG and imaging follow-up, and outcome.

Case	Sex	Age (y)	Operated Side	Grade (McGowan)	Motor Ulnar Nerve Conduction Velocity (m/s) Amplitude (µV)	Time between Surgery and SWE (Months)	Distance Ulnar Nerve/Medial Epicondyle after Transposition (cm)	Shear Elastic Modulus of the Ulnar Nerve (kPa) Non-Operated Side. Elbow Flexion: 0° 45° 90° 120°	Shear Elastic Modulus of the Ulnar Nerve (kPa) Operated Side. Elbow Flexion: 0° 45° 90° 120°
1	male	78	R	2	26 3.9	12	1.2	38.2 30 41.3 82.3	116.2 86.5 66.6 38.9
2	male	41	L	2	35.2 6.9	7	1.0	27.9 38.8 96.8 146.0	150.1 137.0 91.1 58.5
3	female	25	L	2	23 14.4	9	1.1	29.9 92.9 123.2 140.7	260.7 178.6 80.6 60.9
4	male	43	R	1	40 6.8	18	1.0	37.1 86.1 105.5 145.7	165.7 143.3 87.9 52.8
5	female	53	R	2	37 8.1	3	0.49	54.6 56 70.2 157.4	72 56.1 29 49.6
6	female	68	R	2	11 5.2	2	1.23	38 65 88.1 140.7	178.5 126.4 55.7 26.9
7	female	53	L	2	40 9.8	16	0.65	47.8 139.1 144.7 156.3	202.6 146.1 110.7 67.5
8	male	40	L	3	----	3	2.13	39.9 35.2 95.4 111.6	115.8 62.1 72.5 53
9	male	56	L	3	----	3	1.2	35 79.6 122.4 173.5	160.7 70.7 70.2 62.4
10	female	69	R	1	40 5.1	3	1.33	20.6 36.5 57.5 108.5	129.7 100.7 50.5 16.6
11	male	49	L	1	32 6.5	12	2.04	39.4 92.8 115.2 196.3	174.5 151.5 102.8 34.8

## Data Availability

All data generated or analyzed during this study are included in this article. Further enquires can be directed to the corresponding author.

## Abbreviations

CSA	cross-sectional area
ENMG	electroneuromyography
MRI	magnetic resonance imaging
SD	standard deviation
SW	shear-wave
SWE	shear-wave elastography
